# Real Nano “Light Vaccine” Will Benefit to COVID-19 Pandemic Control

**DOI:** 10.1007/s40820-021-00723-2

**Published:** 2021-09-03

**Authors:** Jianshe Yang

**Affiliations:** Shanghai Tenth People’s Hospital, Tongji University School of Medicine, Shanghai, 200072 People’s Republic of China

**Keywords:** SARS-CoV-2, COVID-19, Light, Vaccine, Photothermal conversion, Nanomaterial

## Abstract

This highlight presents a recent technique of “Light Vaccine” for COVID-19 pandemic control. Though this technique has the germicidal advantage to SARS-CoV-2, its shortcomings will limit the wide and in-depth application. We make a perspective of real nano light vaccine, which will play an important role in the prevention and control of COVID-19. Briefly, This flow chart described the MWCNT was fabricated with strong acid and base conditional mixture in order to achieve the p-WCNT (chemical process); then modified with RNA layse and receptor binding domain (RBD) by covalent conjugation and physical absorption to get f-WCNT (functionalization); thereafter, f-WCNT was used in the multi-cell culture system interacting with SARS-CoV-2 to identify the special affinity of f-WCNT to ACE2 labeled alveolar type II cells and the inhibition capacity to SARS-CoV-2. This design, is different from the so called “light vaccine”, has the real function to against SARS-CoV-2 by local cellular temperature-rising through photothermal conversion under the near infrared (NIR) light irradiation, according to the physical and chemical nature of carbon nanotubes, and initiates the immune response consequently.

As reported by the scholars of Fudan University, they, collaborated with Columbia University, developed an equipment named “Light Vaccine”, which can emit pure far-UVC light with 222-nm wavelength, realize a 99.9% germicidal effect to microbes such as virus, bacteria, especially to SARS-CoV-2, and achieve great successes for pandemic prevention and control in recent Tokyo Olympic Games [1].

Professor Brenner, the collaborator of this project, has demonstrated that the 222-nm far-UVC light has promising application in mammalian skin safety [2], airborne-mediated microbial diseases [3], *Staphylococcus aureus* (MRSA) infection of superficial wounds [4] and airborne human coronaviruses [5]. Conventional germicidal ultraviolet light, typically at 254 nm, is effective in this context but, used directly, can be a health hazard to skin and eyes. By contrast, far-UVC light (207–222 nm) efficiently kills pathogens potentially without harm to exposed human tissues. Then, they employed the low doses far-UVC light of 1.7 and 1.2 mJ cm^−2^ inactivated 99.9% of aerosolized coronavirus 229E and OC43, respectively. Considering the human coronaviruses have similar genomic sizes, they found continuous far-UVC exposure in occupied public locations at the current regulatory exposure limit (~ 3 mJ/cm^2^/h) resulted in ~ 90% viral inactivation in ~ 8 min, 95% in ~ 11 min, 99% in ~ 16 min and 99.9% inactivation in ~ 25 min, implied that low-dose-rate far-UVC exposure can potentially safely provide a major reduction in the ambient level of airborne coronaviruses in occupied public locations.

This technique highlights the nonhazardous far UVC light permitting “human–machine coexistence, real-time disinfection”. However, the shortcomings yet remain, which include firstly the low penetrating capacity under so feeble energy and short wavelength, it can only be used to handle surface contamination and useless for the tissues, organs, and cells in the body already infected with the virus; secondly, the accumulation of 30 min exposure in order to reach the 99% germicidal rate, which has not been evaluated for the safety; and thirdly, this is a new technology for effective sterilization using UV light with special wavelength to prevent virus infection, it has nothing to do with vaccine and does not involve any immune responses.

Anyway, this technique in turn reminds us that the simplest measure should be considered for the pandemic control, in that the chemical and biological drugs and vaccines have been demonstrated to be limited efficacy, because of a large amount of asymptomatic patients having robust transmission ability, some convalescent patients emerging re-positive serum viral RNA, and the SARS-CoV-2 variants coming constantly with stronger transmission power and higher lethality.

Nearly all the coronavirus have the common nature that they are irresistible to the relatively higher temperature than 57 °C for 30 min in physiological circumstance. The thermal effect is undoubtedly the simplest and efficient means to kill the virus. What can we apply this advantage effectively?

Partial nanomaterials are outstanding targeted cargoes for various drugs, which play a vital role in the field of precision medicine. Though the nano-vaccines have been reported often, actually the nanomaterials were used only as a carrier or vaccine adjuvant to improve the efficacy, the full potential is not exploited so far. How to expand the scope of the virus elimination, by this simple way, from the body surface and environment to the tissues, organs, cells in the body? A dexterously design is to target modified nanomaterials to the specific areas virus infected, and these nanomaterials can present photothermal conversion effect excited by the biosafety near-infrared light, result in a local high temperature and eventual virus elimination. The most important, this design should initiate the immune responses by special conjugates to up- or down-regulate the competitive binding to ACE2 receptors on the cell membrane. We have proposed to prepare a kind of carbon nanotubes with functions to exert acidification for cytoplasmic and local cellular temperature-rising through photothermal conversion, according to the physical and chemical nature of carbon nanotubes having been well applied to facilitate such a response [6].

By this way, a kind of real light vaccine can be complete rather than an apparatus only can deal with the surface and environment viral contamination.
